# Controversial Interactions of Tacrolimus with Dietary Supplements, Herbs and Food

**DOI:** 10.3390/pharmaceutics14102154

**Published:** 2022-10-10

**Authors:** Miłosz Miedziaszczyk, Aleksander Bajon, Ewelina Jakielska, Marta Primke, Jędrzej Sikora, Dagmara Skowrońska, Ilona Idasiak-Piechocka

**Affiliations:** 1 Department of Nephrology, Transplantology and Internal Medicine, Poznan University of Medical Sciences, 60-355 Poznan, Poland; 2 Student’s Scientific Section of Nephrology and Clinical Transplantology, 61-701 Poznan, Poland

**Keywords:** tacrolimus, interactions, pharmacokinetics

## Abstract

Tacrolimus is an immunosuppressive calcineurin inhibitor used to prevent rejection in allogeneic organ transplant recipients, such as kidney, liver, heart or lung. It is metabolized in the liver, involving the cytochrome P450 (CYP3A4) isoform CYP3A4, and is characterized by a narrow therapeutic window, dose-dependent toxicity and high inter-individual and intra-individual variability. In view of the abovementioned facts, the aim of the study is to present selected interactions between tacrolimus and the commonly used dietary supplements, herbs and food. The review was based on the available scientific literature found in the PubMed, Scopus and Cochrane databases. An increase in the serum concentration of tacrolimus can be caused by CYP3A4 inhibitors, such as grapefruit, pomelo, clementine, pomegranate, ginger and turmeric, revealing the side effects of this drug, particularly nephrotoxicity. In contrast, CYP3A4 inducers, such as St. John’s Wort, may result in a lack of therapeutic effect by reducing the drug concentration. Additionally, the use of *Panax ginseng*, green tea, *Schisandra sphenanthera* and melatonin in patients receiving tacrolimus is highly controversial. Therefore, since alternative medicine constitutes an attractive treatment option for patients, modern healthcare should emphasize the potential interactions between herbal medicines and synthetic drugs. In fact, each drug or herbal supplement should be reported by the patient to the physician (concordance) if it is taken in the course of immunosuppressive therapy, since it may affect the pharmacokinetic and pharmacodynamic parameters of other preparations.

## 1. Introduction

Tacrolimus (TAC, FK506), a well-established calcineurin inhibitor, is the first line treatment agent in the immunosuppression regimen following renal transplantation. However, due to its narrow therapeutic index and within-subject variability, as well as extra-individual variability, it is problematic in terms of the clinical use [1,2]. It should be noted that a tacrolimus overdose may be associated with the occurrence of adverse reactions, such as nephrotoxicity and neurotoxicity, whereas a too low tacrolimus concentration may result in transplant rejection [3]. Consequently, it is essential to understand the impact of the concomitant administration of various substances, such as drugs, dietary supplements and nutrients, on the pharmacokinetics of tacrolimus [1,4]. Changing the bioavailability of a drug constitutes one of the most common clinically relevant interactions between food and drugs [4,5]. Therefore, in order to better comprehend the pharmacokinetics of tacrolimus, knowledge regarding the interactions with dietary supplements or nutrients, particularly at the absorption stage of the drug, is crucial. Furthermore, it has been established that the metabolism of tacrolimus depends on the individual predispositions, such as BMI (body mass index), patient’s age, albumin concentration or hepatic failure [1]. Tacrolimus is mainly metabolised by the cytochrome P450 (CYP450) isoform CYP3A4, which is involved in the metabolism of a number of substances, such as beverages, alcohol, herbs, medications and food [6]. Nevertheless, these substances may be the inducers or inhibitors of the abovementioned enzyme. Thus, the variability of tacrolimus pharmacokinetics may be affected by various factors, including herbs, beverages, alcohol, drugs or the food consumed. As a result, an increase in the concentration of tacrolimus is observed when tacrolimus is used in combination with the inhibitors or substrates of the CYP3A4 isoform, but a decrease in concentration of tacrolimus is also seen when an inducer of this isoform is used. Therefore, interactions between tacrolimus and nutrients may increase, or reduce, the drug effect, contributing to the increase of toxicity, or allograft rejection. The aim of this review is to summarize information with regard to the interactions between tacrolimus and food intake. Additionally, possible adverse side effects have been described for different dietary supplements and nutrients.

## 2. Materials and Methods

The browsing strategy included controlled vocabulary and keywords. The Cochrane, PubMed, and Scopus databases were searched independently by two authors. The main search concept involved a combination of the terms “tacrolimus” AND “interaction” AND “food” or “nutrients” or “ethanol” or “herb” or “herbal” or “dietary supplements”. The inclusion criterion was the data included in the studies related to the interactions selected by the authors. In the recent years, a small number of studies concerning the presented topic have appeared; therefore, we decided not to apply any time criterion when searching the literature for our review. Table 1 shows the methodological procedure.

## 3. Results

### 3.1. Food–Drug Interactions

In addition to numerous well-established drug–drug interactions, Tacrolimus also shows a substantial number of food–drug interactions, with new ones still being found.

The best studied interaction occurs between tacrolimus and grapefruit (fruit or juice). Grapefruit juice contains furanocoumarins: bergamottin and 6′7′-dihydroxybergamottin (6′7′DHB). These compounds may inhibit intestinal CYP3A4, leading to the elevation in tacrolimus blood levels, following oral drug administration. Additionally, grapefruit has an impact on P-glycoprotein (P-gp), where the P-gp inhibition also contributes to an increase in tacrolimus concentration, which may potentially lead to toxicity [7,8,9,10,11,12]. Zhai et al. [7], as well as Liu et al. [11], reported a two-fold growth of blood tacrolimus levels in patients who consumed grapefruit. Therefore, patients should always be advised to avoid both grapefruit and grapefruit juice in the course of immunosuppressive treatment.

Pomelo (*Citrus grandis*) and grapefruit are classified as belonging to the same genus [13,14]; hence, these fruits share similar chemical components. Pomelo contains 6′7′DHB, which inhibits intestinal CYP3A4 [8,13], and other compounds, such as naringin, naringenin, neohesperidin, although their role has not yet been fully evaluated [14]. Egashira et al. [13] reported elevated blood tacrolimus concentration in patients after a one-time ingestion of 100 g of pomelo the day before the blood sample was collected. This finding prompted further investigations, which were conducted on rats. The administration of 100% pomelo juice with tacrolimus resulted in a significant growth in the bioavailability parameters—AUC (area under the concentration–time curve) and Cmax (maximal concentration), in comparison to receiving the drug with water [12]. The same effect was observed when tacrolimus and an extract from pomelo peel were administered simultaneously [14]. Thus, pomelo seems to increase tacrolimus concentration by inhibiting CYP3A4 and P-gp [10,13]. Nevertheless, one study contradicts the interaction between P-gp and pomelo [14]; therefore, further detailed studies in this area are needed.

Another citrus which may cause food–drug interactions is clementine. In 2013, a case of elevated blood tacrolimus levels following the consumption of large amounts of clementines (>1 kg per day) was reported [15]. The impact of clementines was confirmed by a positive de-challenge (a decrease in drug concentration after clementines intake withdrawal) and re-challenge (a repeated increase in tacrolimus blood level following clementines consumption). The mechanism of this interaction is not fully understood, as no furanocoumarins were detected in the clementine juice. It is speculated that clementine juice elevates tacrolimus levels by CYP3A4 inhibition and does not have an impact on P-gp [15]. Due to the scarcity of larger trials, further research is necessary.

Similar effects can be observed after the intake of ginger, turmeric, green tea and pomegranate juice, during treatment with tacrolimus. Possibly, the mechanism of action may be the same and may involve the inhibition of intestinal CYP3A4 and P-gp [8,10,12,16,17,18].

A study on rats revealed a significant growth in the AUC of tacrolimus when co-administered with ginger or turmeric [12]. Nayeri et al. [17] reported a case of acute calcineurin inhibitor nephrotoxicity caused by excessive turmeric intake. The patient had added over 15 spoons of turmeric to his meals daily, which resulted in a three-fold increase in blood tacrolimus level.

Khuu et al. [18] presented a case of pomegranate–tacrolimus interaction following the consumption of popsicles containing pomegranate concentrate. The intake of 1–2 popsicles daily resulted in a two-fold elevation of blood tacrolimus concentration. Interestingly, pomegranate juice contains quercetin, which inhibits CYP3A4. However, the precise mechanism of interaction remains unknown.

Moreover, not only do individual food–drug interactions impact blood tacrolimus levels, as does the timing of meals in relation to the drug administration, as well as general meal composition [19,20,21,22]. Bekersky et al. conducted two studies [21,22] which compared the different timings of tacrolimus intake with its impact on the bioavailability and the effect of low- and high-fat diets. In fact, AUC parameter was significantly higher when tacrolimus was administered following 10 h of fasting in comparison to taking medication 1 h before, immediately after or 1.5 h after breakfast. The groups in which the drug was administered after breakfast showed significantly higher Tmax and lower Cmax levels than groups who received the medication while fasting or 1 h prior to the meal. Nevertheless, the timing of meals does not impact half-life [22]. A study comparing the bioavailability of tacrolimus when administered right after a high-fat or low-fat breakfast revealed that the content of fat in meals does not affect Cmax and AUC parameters. However, Tmax was significantly higher in the group where the subjects ingested the drug after a high-fat breakfast [21]. Additionally, fat consumption leads to slower gastric emptying, which results in a longer time needed for the drug to reach Cmax.

Dave et al. [23] presented a case of a 40-year-old renal transplant patient receiving cranberry supplements as a treatment for recurrent cystitis, which caused a decrease in tacrolimus serum levels. Since the incidence of this ailment is higher in allograft recipients compared to the general population, these patients tend to use cranberry products more frequently [24]. The inhibition of uropathogenic adhesion to uroepithelium is suspected to be the underlying mechanism of cranberry, justifying its common use [25]. Due to the fact that the juice of the fruit affects CYP, the metabolism of drugs administered together with cranberry juice was investigated. The analysis showed an inhibitory influence on CYPs, reducing the metabolism of drugs, such as tacrolimus, and increasing its serum level which, in turn, is in contrast to the data provided in the case report [23].

Table 2 shows the effects of the selected food substances on pharmacokinetics and pharmacodynamics.

### 3.2. Herb–Drug Interactions

Global use of herbal medications, both the products standardized by pharmaceutical companies as well as those used in traditional medicine, are associated with the risk of interactions between the herbal medications and conventional drugs [27,28]. Therefore, a careful insight into tacrolimus treatment administered together with botanical supplements could constitute the basis of a well-targeted therapy.

#### 3.2.1. *Panax ginseng*

*Panax ginseng*, a common component of the traditional medicine, now commonly used worldwide, presents a wide range of indications, including the enhancement of physical and cognitive functions, prevention of cancer, allergies, inflammation and cardiovascular disorders due to its antioxidative and vasorelaxant properties together with antidiabetic effects [27,28,29,30,31,32]. Ginsenosides and their glycosylated intestinal metabolites constitute the most active components in this plant extracts. Consequently, these substances were investigated with regard to the potential modulation of cytochrome P450, glucuronidation reaction and drug transporting proteins. Changes in the serum concentration of the drug depend on the particular drug involved in the drug–herb interaction. Some preclinical tests provide data about the potential inhibition of CYP2D6 and P-gp, as well as CYP3A induction, although it is vital to bear in mind that in the majority of cases these changes are not clinically significant [28,33,34]. As a herbal product, *Panax ginseng* comprises numerous ginsenosides, as well as other untested components, which could be inconsistent between single plants, formulations and ready products. Therefore, all of the abovementioned factors could alter the metabolism of tacrolimus. Although there are no conclusive tests excluding the impact of ginsenosides on the metabolism of tacrolimus, the risks of such an interaction are rather low; nevertheless, they should not be neglected [28,29,33,34].

Nephrotoxicity is one of the main causes of chronic allograft dysfunction and allograft failure in transplant recipients [35,36]. The main associated factor seems to be oxidative stress and the formation of reactive oxygen species (ROS) generated by calcineurin inhibitors (CNIs), including tacrolimus [36,37]. Klotho is an anti-ageing gene involved in that process, expressed mainly in the kidneys. The overexpression of that gene results in the extended lifespan in mice, and additionally, reverses the state of detrimental mutations in the nucleotide structure of Klotho, thus leading to premature ageing-like effects and decreased lifespan [38]. Lim et al. [39] investigated the potential of *Panax ginseng* to reduce TAC-induced oxidative stress, associated with the signalling between Klotho and the related of antioxidant pathways: phosphatidylinositol 3-kinase(PI3K)/serine-threonine kinase Akt(AKT)/forkhead box protein O3a(FoxO3a). Murine models suggest that the combination of tacrolimus with *Panax ginseng* promotes Klotho and its antiaging signalling, potentially via the inhibition of PI3K/AKT-induced FoxO3a phosphorylation and resulting in the enforcement binding of FoxO3a to the manganese superoxide dismutase (MnSOD) promoter region. Thereby, such a combination amplifies the production of MnSOD mRNA by enzyme expression enhancement in the mitochondria. Furthermore, an increased MnSOD concentration improves the detoxification of ROS to hydrogen peroxide and diatomic oxygen, consequently attenuating tacrolimus-induced oxidative injury in the mitochondria [39,40]. A histological examination of the renal cortex in mice treated with TAC revealed micro-injuries, atrophy and tubulointerstitial fibrosis, whereas an additional usage of *Panax ginseng* significantly reduced such lesions [39]. Interestingly, prolonged tacrolimus-induced oxidative stress causes adaptive changes in the cell, such as the formation of multiple autophagosome. However, the increased population of autophagosomes is not efficiently degraded due to the impaired autophagic clearance (lysosomal degradation and autophagosome fusion with lysosomes). The overflow of these structures results in the autophagic cell death. Therefore, treatment with *Panax ginseng* improves the autophagic clearance function by enhancing lysosomal function and autophagosome fusion with lysosomes [41,42]. Due to the excess of ROS in the entire body during tacrolimus therapy, other adverse effects are likely to occur, including direct oxidative toxic effects on the pancreatic beta cells, which may result in diabetes mellitus in the population of recipients [41,43,44]. Nevertheless, further research will elucidate whether the reduction of oxidative stress by *Panax ginseng* is sufficient to prevent such complex changes. Furthermore, Doh KC et al. [45] demonstrated the protective effects of *Panax ginseng* against the side effects of cyclosporine, where such an additional treatment was associated with a decreased blood urea nitrogen, interstitial inflammation, fibrosis and apoptosis. Another beneficial activity of *Panax ginseng* is the favourable modulation of immune homeostasis [34,46]. This herb is well documented to ameliorate the conditions of autoimmune patients [47,48,49]. In fact, it regulates Th17 cells population and reciprocally promotes Treg cells by inhibiting the phosphorylation of signal transducer and activator of transcription 3 phosphorylation (STAT3) pathways, thus establishing Th17/Treg balance [47]. In view of all the available data regarding *Panax ginseng* supplementation while receiving CNIs, its role in mitigating the chronic side effects of tacrolimus is worth emphasizing. However, the effect of Panax ginseng on CYP-mediated drug metabolism or through other pathways remains inconclusive.

#### 3.2.2. Green Tea

Oriental tea infusion has become popular across the globe in the recent decades. Green tea has been shown to inhibit CYPs as well as P-glycoprotein, whose role is to remove various foreign substances from cells in the body. This reducing effect, in turn, should result in an increase in plasma drug level [50,51,52]. However, the aforementioned inhibitory effect is not shared by all the components of botanical supplements and may differ between particular substances [53,54]. In one case report [18], researchers observed an increase in tacrolimus levels following green tea ingestion, and its normalization after the tea was discontinued. Further investigation showed that the patient represented a “poor metabolizer”. This case proved the importance of careful monitoring of this group of recipients, particularly considering the narrow therapeutic index of TAC. In contrast, murine models show a reduction in the Cmax and AUC of tacrolimus following the co-administration of specific catechins extracted from green tea [16]. However, polymerase chain reaction and Western blot showed a reducing effects of catechins on the expression of drug-metabolizing enzymes, drug transporters and the upregulation of nuclear receptors. This inconsistency needs to be further explored in the subsequent studies [55,56,57]. Despite the pharmacokinetic alteration following green tea intake, the protective properties of this herb constitute its main advantage. Commonly known antioxidative properties of green tea can reduce the nephrotoxic effects of tacrolimus, as well as play a role in preventing the development of cardiovascular diseases. Moreover, tea extracts prevent apoptotic cell death via modulation of mitochondrial cytochrome c release and modulation of caspase activation and also decrease ROS, NO and other detrimental substances in a dose- and time-dependent manner [58,59,60,61,62]. Theaflavins and catechins are active ingredients of the plant, and they do not demonstrate as high activity as the complete green tea extracts when tested separately [61]. All the reviews investigated only green tea, not black tea created by the fermentation of natural products; thus, the interaction with tacrolimus may be different. However, there is a need to explore various herbal–drug interactions in the future. Much of the currently available data are derived only from case reports conflicting with the established mechanisms of action, indicating other possible mechanisms. In 2011, a case of a 1.5–2-fold increase of tacrolimus concentration was observed after the ingestion of green tea [18]. Nevertheless, authors did not provide precise data concerning the type of green tea and the amount consumed.

#### 3.2.3. *Echinacea purpurea*

Users of immunosuppressants struggle with multiple infections, frequently resistant to the commonly used antibiotics. Therefore, patients often resort to taking various natural supplements, such as the already mentioned cranberry, or Echinacea; hence, it is vital to note the possible interactions of all the substances used by the patients. Nevertheless, the literature data provide conflicting findings. The study by Gorski et al. [63] reported evidence of a reduced oral clearance of substrates of CYP1A2, although not the oral clearance of substrates of CYP2C9 and CYP2D6 when co-administered with *Echinacea purpurea*. In fact, Echinacea selectively modulates the catalytic activity of CYP3A at hepatic and intestinal sites. On the other hand, data from different studies suggested that no significant influence on metabolic enzymes was observed [64,65].

#### 3.2.4. *Hypericum perforatum*

St. John’s Wort (*Hypericum perforatum*; SJW) is a herbal supplement commonly used for the treatment of mild to moderate depression, commonly observed in transplant patients [66]. The most significant ingredients of SJW are hyperforin and hypericin. Hyperforin has an antidepressant effect—SJW in doses 180–1800 mg (4–5% hyperforin content- averaging about 40 mg per dose) but also accounts for drugs interactions [67]. Hyperforin induces intensified the expression of CYP3A4 in hepatocytes and P-glycoprotein through activating PXR (pregnane X receptor), whereas hypericin induces intestinal P-glycoprotein [68,69]. In a clinical trial, short-term (3–4 days, 900 mg/day) SJW extract administration no influence on CYP3A4 was found, although long-term usage (14 days, 900 mg/day) could induce the expression of CYP3A4 in the liver and intestine cells [70]. Tacrolimus is metabolized by CYP3A4 and is a substrate of P-glycoprotein; therefore, the abovementioned mechanisms contribute to a severe decrease (>50%) in the bioavailability of tacrolimus [53,71]. According to another clinical trial, the administration of SJW extract to renal transplant patients at a dose of 600 mg/day for 14 days decreased the AUC of tacrolimus by 57.8%, which resulted in an increased risk of transplant rejection [70]. An important practical aspect of receiving St. John’s Wort is that tea does not contain significant amounts of hyperforin, whereas the concentrations present in the hydroalcoholic extract are sufficient to cause drugs interactions. Nevertheless, the influence on the activity of the enzyme may differ depending on the CYP isoform, dose, administration schedule or the origin of the herb [70,72,73]. A hyperforin dose up to 1 mg/day does not interact with other substances, and thus can be safely administered with tacrolimus [67].

Table 3 shows the effects of the herbal substances on pharmacokinetics and pharmacodynamics.

### 3.3. Dietary Supplements Interactions

#### 3.3.1. *Schisandra sphenanthera*

A *Schisandra sphenanthera* ethanol extract (SchE) is a herbal medicine also referred to as a Wuzhi capsule, or a Wuhzi tablet, which has been used in the traditional Chinese medicine for thousands of years in cases of hepatitis, hepatic and renal insufficiency, menstrual dysfunction, neurosis and others [74,75,76,77]. It is often prescribed in Chinese transplant patients for tacrolimus-induced hepatitis treatment as a liver-protective agent [77,78]. Studies have shown that the co-administration of Wuhzi tablets and tacrolimus may significantly increase tacrolimus blood concentration. Schisantherin A (STA) and schisandrin A (SIA), the main active agents of SchE, inhibit CYP3A4/5 by both reversible inhibition and the permanent loss of enzyme activity through its destruction [76,79]. Other SchE components, schisandrin B and gomisin A, were found to inhibit P-glycoprotein, whereas gomisin C competitively inhibited and irreversibly deactivated CYP3A [78]. Tacrolimus is metabolized by CYP3A isoenzymes and undergoes P-glycoprotein-mediated transport; therefore, an interaction should be expected [4,76,80]. Tacrolimus blood concentrations increased 1.57–4.66-fold following SchE administration [76]. Moreover, parameters, such as the AUC, AUMC and Cmax of tacrolimus, were reported to increase significantly, although in terms of CL/F and V/F a significant decrease was observed [78,81]. The SchE effect on Cmax and AUC in rats was dose-dependent and the maximum mean plasma concentration of tacrolimus was achieved at 450 mg/kg [77]. Interestingly, a higher SchE dose did not result in an increase in tacrolimus concentration. Furthermore, a single SchE dose administration resulted in a higher mean plasma concentration and AUC compared to a multiple SchE dosing [77]. Despite the remarkable increase in the blood AUC of tacrolimus, no significant changes were found in rat tissue concentrations [82]. Moreover, studies on CYP3A5 polymorphism showed that SchE effect was more pronounced in the CYP3A5 expressers compared to the non-expressers [79,81,83]. An increase in tacrolimus concentration was lower, although still significant when combined with a long-term prednisone treatment [84]. Xin et al. reported lower mean creatinine levels and higher mean BUN levels in healthy volunteers after SchE and tacrolimus co-administration [78]. In terms of liver function, ALT and AST levels significantly decreased following 6-month SchE treatment in renal transplant patients receiving tacrolimus [85]. SchE administration in renal transplant recipients not only did not deteriorate graft function but was found to have a protective effect on its survival [81]. Side effects, such as hyperkalaemia or fever, were reported after SchE and tacrolimus co-administration and were accounted for as a result of an increased tacrolimus blood concentration [78,86]. Several population pharmacokinetic models have been developed to support the individualization of tacrolimus dosing for different groups: renal transplant recipients [81,83,85,87,88], liver transplant recipients [89,90], cardiac transplant recipients [91,92], patients with refractory nephrotic syndrome [93,94], membranous nephropathy [95,96], lupus nephritis [97,98] or myasthenia gravis [99]. SchE may significantly increase tacrolimus bioavailability and reduce its therapeutic costs by 40–60%, nevertheless, authors highly recommend blood tacrolimus concentration monitoring while co-administering with SchE [78,79,82,85].

#### 3.3.2. Melatonin

One of the side effects involving tacrolimus is nephrotoxicity. The mechanism of kidney damage relies on such factors as renal blood flow restriction via the contraction of afferent and efferent glomerular arterioles, and an increase in the formation of reactive oxygen species. In fact, they frequently result in endothelial cell failure and lead to tubulointerstitial nephritis [100]. According to findings of trials on rodents, melatonin may have an alleviating influence on the pro-oxidative action of tacrolimus due to its strong antioxidant effects. The animals treated with tacrolimus with melatonin presented reduced laboratory parameters, such as malondialdehyde (MDA) (a marker of lipid peroxidation), tumour necrosis factor-alpha (TNF-α) and Interleukine-6 (IL-6), with a simultaneously higher Nitric Oxide (NO) level than the animals treated with tacrolimus without melatonin. Moreover, some findings suggest that the concomitant tacrolimus–melatonin therapy may potentially prevent acute kidney injury (AKI) resulting from ischemia-reperfusion injury more effectively than each drug separately [101]. Thus, the abovementioned data may suggest that the therapy involving tacrolimus in combination with melatonin may present less severe side effects [102,103].

Table 4 shows the effects of the selected substances on pharmacokinetics and pharmacodynamics.

## 4. Conclusions

In conclusion, patients starting treatment with tacrolimus should always be advised not to consume grapefruit and pomelo, as well as clementine, pomegranate, ginger, turmeric and green tea in excessive amounts. Furthermore, tacrolimus should be administered according to a regular regimen, accounting for the timing of breakfast and drug administration. Meals ought to have a consistent composition.

All herbs contain thousands of different elements, which may affect the pharmacokinetics of TAC. Although interactions can be problematic, it is essential to address the factors which influence the pharmacokinetics and pharmacodynamics parameters of tacrolimus: dosage, differences involved in herbal medicines production, interaction with other drugs, comorbidities, and patients’ age and their metabolic predispositions. In view of the aforementioned factors, as well as the narrow therapeutic range of tacrolimus, the careful monitoring of patients, particularly the individuals referred to as “poor metabolizers”, is crucial. However, the protective properties of *Panax ginseng*, green tea, *Schisandra sphenanthera* and melatonin may be crucial in mitigating the side effects of tacrolimus and, consequently, improving the long-term condition of the graft, as well as the health of the recipients. The aqueous solution of St. John’s Wort does not contain significant amounts of hyperforin, whereas the concentrations present in the hydroalcoholic extract are sufficient for the enzymatic induction, e.g., the CYP3A4 form. Hence, the combined administration of SJW extract with tacrolimus is contraindicated.

Alternative medicine is an appealing treatment option for patients. Therefore, modern healthcare should emphasize the potential interactions between herbal medicines and synthetic drugs. Each drug or herbal supplement should be reported by the patient to the physician (concordance) if it is taken in the course of immunosuppressive therapy, since it may affect the pharmacokinetic and pharmacodynamic parameters of other preparations.

## Figures and Tables

**Table 1:** The methodological procedure.

Databases: Cochrane, PubMed, Scopus		
Stage	Action	Results
I	key words (“tacrolimus” AND “interaction” AND “food” or “nutrients” or “ethanol” or “herb” or “herbal” or “dietary supplements”)	125
II	independent verification and inclusive research by two authors inclusion criteria: the agents selected by the authors: grapefruit, pomelo, clementine, ginger, turmeric, pomegranate juice, *Panax ginseng*, green tea, St. John’s Wort, cranberry, *Echinacea purpurea*, melatonin and *Schisandra sphenanthera* extract	105
III	due to the small number of studies in recent years, the time criterion has not been applied	105

**Table 2:** The effects of the selected food substances on pharmacokinetics and pharmacodynamics [7,8,11,12,13,15,18,23,26].

Food	Active Component	Relevant Metabolizing Enzymes/Transporters/Mechanism of Actions	Pharmacokinetics of Tacrolimus	Adverse Effects
Grapefruit	Furanocoumarins: bergamottin 6′7′-dihydroxybergamottin	Inhibition of intestinal CYP3A4 Inhibition of P-gp	Elevation of TAC serum level	Aggravation of TAC toxicity
Pomelo	Naringin, Naringenin, Neohesperidin 6′7′-dihydroxybergamottin	Inhibition of intestinal CYP3A4 Inhibition of P-gp	Elevation of TAC serum level	Aggravation of TAC toxicity
Clementine	-	Inhibition of CYP3A4	Elevation of TAC serum level	Aggravation of TAC toxicity
Turmeric	-	-	Elevation of TAC serum level	Aggravation of TAC toxicity
Ginger	-	-	Elevation of TAC serum level	Aggravation of TAC toxicity
Pomegranate	Quercetin	Inhibition of CYP3A4	Elevation of TAC serum level	Aggravation of TAC toxicity
Cranberry	-	Influence on CYPs	Inconclusive data	Inconclusive data

TAC—tacrolimus; P-gp—P-glycoprotein.

**Table 3:** The effects of the selected herbal substances on pharmacokinetics and pharmacodynamics [28,33,34,50,51,52,61,67,68,69,70].

Herb	Active Component	Relevant Metabolizing Enzymes/Transporters/Mechanism of Actions	Pharmacokinetics of Tacrolimus	Impact on Organism
*Panax ginseng*	Ginsenosides Glycosylated intestinal metabolites	Poor inhibition of CYP2D6; Poor inhibition of P-gp; Poor induction of CYP3A; Promotion of Klotho signalling pathway	Poor increase or lack of impact on TAC serum level	Poor or no aggravation of TAC toxicity, a decrease in oxidative stress with a reduction in TAC-induced nephrotoxicity
*Green tea*	Catechins Theaflavins	Inhibition of CYPs; Inhibition of P-gp; Promotion of Klotho signalling pathway	Inconclusive data	A decrease in oxidative stress with a reduction in TAC-induced nephrotoxicity
*Echinacea purpurea*	-	Inconclusive data	Inconclusive data	Inconclusive data
*Hypericum perforatum*	Hyperforin Hypericin	Induction of CYP3A4; Induction of P-gp	A decrease in TAC serum level	Increased risk of transplant rejection

TAC—tacrolimus; P-gp—P-glycoprotein.

**Table 4:** The effects of the selected substances on pharmacokinetics and pharmacodynamics [76,77,78,79,100,101].

Dietary Supplement	Active Component	Relevant Metabolizing Enzymes/Transporters/Mechanism of Actions	Pharmacokinetics of Tacrolimus	Impact on Organism
*Schisandra sphenanthera*	Schisantherin A (STA); Schisandrin A (SIA); Gomisin C; Schisandrin B; Gomisin A	Inhibition of CYP3A4; Inhibition of CYP3A5; Inhibition of P-gp	Elevation of TAC serum level	Exacerbation of TAC toxicity; Protective effect on the liver
Melatonin		-	-	A decrease in oxidative stress, with a decreased risk TAC-induced nephrotoxicity

TAC—tacrolimus; P-gp—P-glycoprotein.

## Data Availability

Not applicable.
